# Folic Acid Self-Assembly Enabling Manganese Single-Atom Electrocatalyst for Selective Nitrogen Reduction to Ammonia

**DOI:** 10.1007/s40820-021-00651-1

**Published:** 2021-05-12

**Authors:** Xuewan Wang, Dan Wu, Suyun Liu, Jiujun Zhang, Xian-Zhu Fu, Jing-Li Luo

**Affiliations:** 1 Shenzhen Key Laboratory of Polymer Science and Technology, Guangdong Research Center for Interfacial Engineering of Functional Materials, College of Materials Science and Engineering, Shenzhen University, Shenzhen, 518060 People’s Republic of China; 2 Institute for Sustainable Energy, College of Sciences, Shanghai University, Shanghai, 200444 People’s Republic of China

**Keywords:** Folic acid self-assembly, N-doped carbon sheet, Manganese single-atom catalyst, Electrocatalysis, Nitrogen reduction

## Abstract

Efficient and robust single-atom catalysts (SACs) based on cheap and earth-abundant elements are highly desirable for electrochemical reduction of nitrogen to ammonia (NRR) under ambient conditions. Herein, for the first time, a Mn–N–C SAC consisting of isolated manganese atomic sites on ultrathin carbon nanosheets is developed via a template-free folic acid self-assembly strategy. The spontaneous molecular partial dissociation enables a facile fabrication process without being plagued by metal atom aggregation. Thanks to well-exposed atomic Mn active sites anchored on two-dimensional conductive carbon matrix, the catalyst exhibits excellent activity for NRR with high activity and selectivity, achieving a high Faradaic efficiency of 32.02% for ammonia synthesis at  − 0.45 V versus reversible hydrogen electrode. Density functional theory calculations unveil the crucial role of atomic Mn sites in promoting N_2_ adsorption, activation and selective reduction to NH_3_ by the distal mechanism. This work provides a simple synthesis process for Mn–N–C SAC and a good platform for understanding the structure-activity relationship of atomic Mn sites.

**Graphic Abstract:**

**Supplementary Information:**

The online version contains supplementary material available at 10.1007/s40820-021-00651-1.

## Highlights

- A manganese single-atom catalyst is developed via a facile folic acid self-assembly strategy.
- The catalyst exhibits outstanding activity and selectivity for electrochemical reduction of nitrogen to ammonia (NRR).
- Electrocatalytic mechanism of Mn–N_3_ site for NRR is unveiled by a combination of experimental and computational study.

**Supplementary Information:**

The online version contains supplementary material available at 10.1007/s40820-021-00651-1.

## Graphical abstract

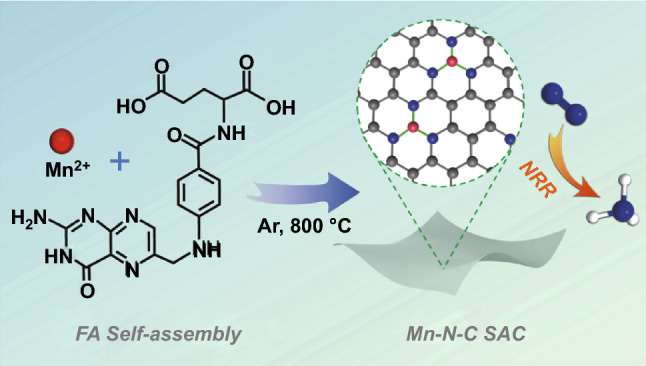

## Introduction

Using atmospheric nitrogen (N_2_) as a feedstock to artificially produce ammonia (NH_3_) is central to fertilizer industry and offers a potential carbon–neutral and energy-dense hydrogen carrier for future energy technologies [1, 2]. Currently, the direct use of N_2_ heavily relies on the energy-intensive Haber–Bosch process, a century-old industrial process coming at the cost of safety issues and CO_2_ emission effect. Electrochemical reduction of N_2_ (NRR, N_2_ + 6e^−^ + 6H^+^  → 2NH_3_) is a promising nitrogen-fixation system that can sustainably operate under mild conditions [3, 4]. However, the efficient generation of NH_3_ is difficult due to the sluggish cleavage of chemically inert N≡N bond [2, 3, 5, 6]. Moreover, NRR involves multiple intermediates and needs to compete with the hydrogen evolution reaction (HER) in aqueous solution, which makes limited Faradaic efficiency (FE) for NH_3_ [7–14]. Electrocatalysts based on precious metals like Ru and Rh have been experimentally and theoretically explored to present favorable NRR activity [6, 15–18]. From practical standpoint, it is significant to develop robust and selective electrocatalysts for NRR from cost-effective metals. Manganese (Mn) has attracted more and more attention due to its low-cost, earth-abundant and eco-friendly nature. However, NRR catalyzed by Mn-based heterogeneous electrocatalysts has been rarely reported due to the poor activity and ammonia selectivity.

Recently, single-atom catalysts become a rising star for a range of electrocatalytic applications due to the integrated merits of maximized atom utilization efficiency, tailorable metal active sites and distinct catalytic properties from their nanoparticle equivalents [19–23]. However, the thermodynamically unstable nature of single metal atoms poses challenges for preparing stable SACs. To successfully engineer SACs, suitable precursors (including metal and supporting materials), effective synthetic strategies and intriguing metal-support interactions are three important considerations, which are also intimately correlated with the exotic geometric and electronic structures of SACs [22, 24, 25]. As a representative, Mn single-atom catalysts (Mn SACs) with Mn^δ+^-N_x_ sites have been developed and proved to be highly active for CO_2_ reduction and oxygen reduction; however, they have seldom been explored for NRR [26–30]. Moreover, the fabrication of Mn SAC with increased dispersion intensity of Mn atoms remains a grand challenge because Mn atoms are easily oxidized and tend to aggregate into oxide /carbide species during the thermal treating process even at a low content [27, 31, 32].

Herein, we develop a folic acid (FA) self-assembly strategy to fabricate a new Mn SAC with exclusive Mn–N_3_ single-atom sites on ultrathin N-doped carbon sheets (denoted as Mn–N–C SAC). The engineered Mn–N–C SAC shows remarkable electrocatalytic performance for triggering NRR to ammonia, with a maximum ammonia faradaic efficiency (FE_NH3_) of up to 32.02% and a desirable yield rate of 21.43 μg h^−1^ mg^−1^_cat_. This demonstrates the new synthesis of Mn SAC for enhanced NRR. In-depth theoretical analysis unveils the intriguing electrocatalytic properties of Mn–N_3_ active sites.

## Experimental Section

### Synthesis of the Catalysts

FA powder (110 mg) was dispersed in 13.5 mL of deionized water–ethanol mixed solution (v/v = 8:5.5), followed by the addition of MnCl_2_·4H_2_O (49 mg). The resulted solution was ultrasonicated for 30 min and then hydrothermally treated at 140 °C for 2 h. The obtained FA-Mn NS with a yield of 34.3% with respect to the amount of FA was collected, ultrasonicated, washed with deionized water for 5 times and then freeze-dried. FA-Mn NS precursor was transferred into crucible for pyrolysis. The pyrolysis process was proceeded under Ar atmosphere and kept at 800 °C for 2 h, leading to the Mn–N–C SAC sample with a yield of 28.6% for use without any further treatment. N-doped carbon nanosheets were synthesized following the same procedure except for the addition of metal source.

### Material Characterization

Morphological information was obtained from FESEM (HITACHI SU8010) and TEM (JEOL-F200). The atomic metal dispersion was confirmed by HAADF-STEM images, EELS spectra and EDS mappings taken from STEM (Titan Cubed Themis G2 300). XRD patterns were collected using Bruker AXS D8 Advance instrument with Cu Kα radiation (λ = 1.5406 Å). XPS experiments were performed on Thermo Scientific K-Alpha + spectrometer. Specific surface area was measured on a Quantachrome AUTOSORB-1 system. ICP-AES measurements were performed on ICAP 7000 SERIES to determine the metal loading of the catalysts. The atomic coordination environment of the catalysts was investigated by synchrotron Spherical Grating Monochromator (SGM) beamline and Very Sensitive Elemental and Structural Probe Employing Radiation (VESPERS) beamline of Canadian Light Source. The data were analyzed by ATHENA software and fitted by IFEFFIT program.

### Electrochemical Measurements

The electrochemical tests were carried out in a typical H-type cell separated by Nafion 211 membrane. To prepare the catalyst ink, the catalyst sample (5 mg) was suspended in a mixture solution of isopropanol (950 μL) and Nafion (5 wt%, 50 μL), followed by an ultrasonication treatment for 2 h. Then the obtained ink was dropped onto the carbon fiber paper to prepare the working electrode, with a loading content of 0.25 mg cm^−2^. Ag/AgCl (3.0 M KCl) and platinum plate (1 × 1 cm^2^) were used as the reference electrode and counter electrode, respectively. The NRR tests were performed in 0.1 M NaOH solution. All the potentials in this work were calibrated to RHE, *E* (RHE) = *E* (Ag/AgCl) + 0.210 + 0.059 pH.

Before the electrochemical measurements, Nafion membrane was pretreated with 3 wt% hydrogen peroxide at 80 °C for 1 h, rinsed with deionized (DI) water and then soaked in DI at 80 °C for another 1 h. To remove the possible N contaminates, all the feeding gases including ^14^N_2_ (99.999%), ^15^N_2_ (99%) and Ar (99.999%) were subsequently passed through an alkaline trap of 0.1 M NaOH and an acidic trap of 0.05 M H_2_SO_4_. Before the experiment, the electrolyte was saturated with the purified Ar or N_2_ for at least 30 min and the bubbled gas was maintained during the experiments. Linear sweep voltammetry (LSV) measurements were performed at a scan rate of 10 mV s^−1^. The chronoamperometry tests were conducted at a constant potential of −0.25, −0.35, −0.45, −0.55, and −0.65 V vs. RHE for 2 h.

### Determination of Ammonia

To analyze the yield rate and Faradaic efficiency of ammonia, the produced NH_3_ in the electrolyte was detected by the typical indophenol blue method [33, 34]. All the yield rate and Faradaic efficiency are calculated from the average values of three repetitive measurements. In detail, 2 mL of NRR-obtained electrolyte was firstly mixed with 1.25 mL of 0.625 M NaOH containing salicylic acid (0.36 M) and sodium citrate (0.17 M), followed by a subsequent addition of 75 μL of NaClO (available chlorine 4.0 wt%) and150 μL of C_5_FeN_6_Na_2_O (10 mg mL^−1^). The mixed solution was incubated for 2 h under ambient condition and then the formed indophenol blue was measured by UV–vis spectrophotometer at the absorption wavelength of 658 nm. To calculate the concentrations of the ammonia, a calibration curve was constructed from the standard NH_4_Cl in 0.1 M NaOH.

### Determination of Hydrazine

The hydrazine in the electrolyte was examined by the method of Watt and Chrisp [35]. A mixture of para(dimethylamino)-benzaldehyde (4.0 g), HCl (37%, 24 mL) and ethanol (200 mL) was used as a color reagent. 2 mL of the electrolyte was mixed with 2 mL of the color reagent. After a 30 min incubation at ambient condition, the mixed solution was measured at 458 nm. The calibration curve was constructed using standard hydrazine monohydrate solution, which was prepared at different concentrations in 0.1 M NaOH.

### ^15^N_2_ Isotope Labeling Experiments

Isotope labeling test was performed using the above-mentioned method with the feeding gas been changed to ^15^N_2_ enriched gas (99%). The formed ^15^NH_3_ in the electrolyte was examined by using ^1^H NMR spectroscopy (nuclear magnetic resonance, 600 MHz).

### FE and Yield Rate Calculations

The FE and mass-normalized yield rate of NH_3_ are calculated according to the formulas as follows:

1 $${\text{FE }} = \, (3 \times F \times c \times V) \, / \, Q$$

2 $${\text{Yield rate }} = \, 17 \times c \, \times \, V \, / \, (t \times m)$$

where *F* is the Faraday constant (96,485 C mol^−1^), *c* is the concentration of NH_3_, *V* is the volume of the electrolyte, *Q* is the total charge passed through the electrode, *t* is the electrolysis time (2 h) and *m* is the total mass of the catalyst.

### DFT Calculations

Vienna Ab Initio Package (VASP) is employed to conduct the density functional theory (DFT) calculations within the generalized gradient approximation (GGA) using the PBE formulation [36–38]. Projected augmented wave (PAW) potentials [39, 40] are selected to describe the ionic cores and the valence electrons were taken into account with a plane wave basis set being used (kinetic energy cutoff, 400 eV). Under the Gaussian smearing method, partial occupancies of the Kohn − Sham orbitals are allowed with a width of 0.05 eV. The self-consistence of the electronic energy is reached until the energy change smaller than 10^−5^ eV. The convergence of the geometry optimization is achieved till the force change smaller than 0.02 eV Å^−1^. Grimme’s DFT-D3 methodology is used to describe the dispersion interactions [41].

The equilibrium lattice constant of hexagonal graphene monolayer unit cell separated by vacuum in depth of 15 Å is optimized to be a = 2.468 Å, with a 15 × 15 × 2 Monkhorst–Pack k-point grid being used for Brillouin zone sampling. Then, it is used to construct a graphene monolayer supercell model with *p* (5 × 5) periodicity in the x and y directions. To separate the graphene monolayer from its periodic duplicates, the supercell model is separated by vacuum in depth of 15 Å. A Mn–N_3_ moiety is embedded into the graphene model to mimic Mn SAC. During structural optimizations, the Γ point in the Brillouin zone is used for k-point sampling, and all atoms are allowed to fully relax.

The free energy of a gas phase molecule and the adsorbates on the constructed model are calculated according to the formula: *G* = *E* + ZPE – *TS*, where *E* is the total energy, ZPE is the zero-point energy, *T* is the temperature in kelvin (298.15 K used here), and *S* is the entropy.

## Results and Discussion

### Synthesis and Characterizations of Mn–N–C SAC

**Fig. 1:**
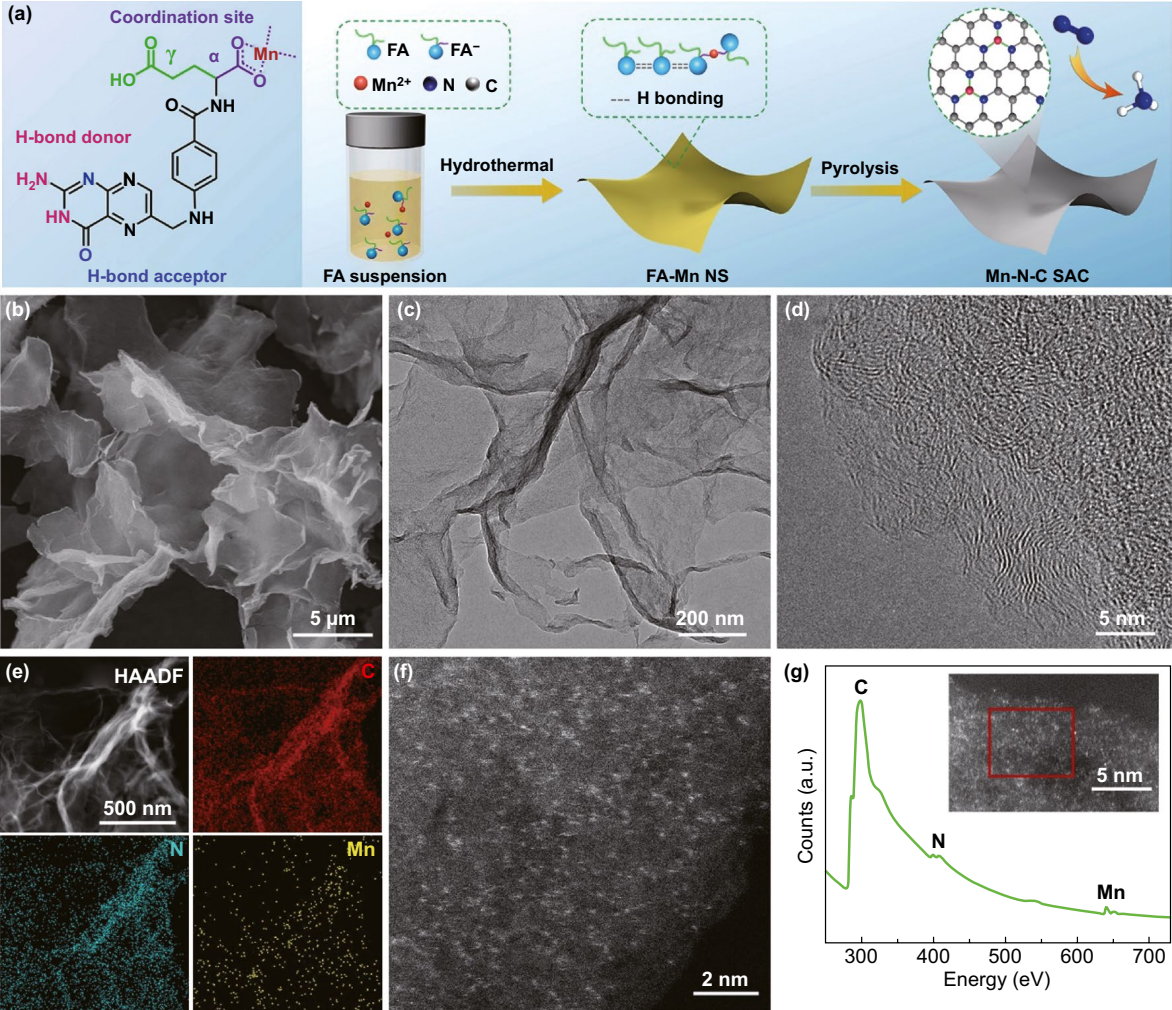
**a** Schematic of the synthetic procedure of Mn–N–C SAC. **b** SEM image of FA-Mn NS. **c** TEM and **d** bright-field STEM images of Mn–N–C SAC. **e** HAADF-STEM image of Mn–N–C SAC and the corresponding EDS elemental mapping images. **f** Aberration-corrected STEM image and **g** the corresponding EELS curves of Mn–N–C SAC

The multistep synthesis of Mn–N–C SAC is schematically depicted in Fig. 1a. Specifically, FA powder is suspended and then hydrothermally dissolved in a mixed solvent of ethanol and water. In this process, FA molecules partially dissociate at α-carboxyl group (pK_α_ ≈ 3.38), which leads to a measured pH value of 4.25 (< pK_γ_ ≈ 4.98 of γ-carboxyl group) for initial FA suspension [42, 43]. The dissociated molecules can efficiently chelate Mn ions. Synchronously, the dissolved molecules self-assemble into unusual supramolecular nanosheets by means of complementary hydrogen bonding interaction at pteridine groups [44]. The synergy of FA partial dissociation and self-assembly simply integrates a controllable amount of Mn ions into the FA supramolecular nanosheets. Details of the intermolecular interaction and metal–ligand coordination can be found in Fig. S1. Thereafter, FA-Mn supramolecular nanosheets (denoted as FA-Mn NS) are pyrolyzed at 800 °C under Ar atmosphere to obtain Mn–N–C SAC. During the pyrolysis, the chelated Mn ions are physically isolated by undissociated FA molecules and then anchored onto the resulted carbon matrix by in situ formed C-N_x_ species, which can effectively prevent them from aggregation.

Field-emission scanning electron microscopy (SEM) and high-resolution transmission electron microscopy (HR-TEM) images reveal the ultrathin and amorphous structure of as-synthesized FA-Mn NS (Figs. 1b and S2). The thickness of the nanosheets is measured to be 2.5–3 nm by atomic force microscopy (AFM) (Fig. S3). X-ray photoelectron spectroscopy (XPS) analysis proves the formation of FA-Mn NS that exhibits consistent structural components with FA except for Mn (Fig. S4). The content of Mn in the nanosheets is measured to be 0.57 wt% by inductively coupled plasma-atomic emission spectrometry (ICP-AES). Then, the metal–organic precursor is directly transformed into Mn–N–C SAC by thermal pyrolysis. As shown by TEM image (Fig. 1c), Mn–N–C SAC preserves the ultrathin structure with rough surfaces. AFM analyses in Fig. S5 demonstrate that Mn–N–C SAC has an average thickness of ~ 1.5 nm, verifying the graphene feature of the catalyst. Such nanosheet structure endows the catalyst with abundant well-exposed active sites for NRR. Further bright-field scanning TEM (STEM) inspection clearly illustrates that the carbon matrix of the catalyst is structured with disordered carbon lattices (Fig. 1d), indicating a low graphitization degree. The high angle annular dark-field (HAADF)-STEM further confirms the ultrathin feature of Mn–N–C SAC and excludes the presence of detectable Mn aggregates (Fig. 1e). The associated EDS elemental mapping images display an even distribution of C, N and Mn elements across the whole carbon sheet. The homogeneous and atomistic dispersion of Mn throughout the carbon matrix can be directly monitored as monodisperse white spots by the aberration-corrected STEM (Fig. 1f). The mass loading of Mn is determined to be as high as 2.82 wt% by ICP-AES, comparable to the metal loading of typical transition metal (*e.g.*, Co, Fe or Ni)-N–C SACs [45–47]. In the electron energy-loss spectroscopy (EELS) curve (Fig. 1g), evident C, N and Mn signals are measured from the inset selected area, which implies the likely Mn–N interactions. N_2_ adsorption–desorption isotherm in Fig. S6a demonstrates that Mn–N–C SAC has a Brunauer–Emmett–Teller (BET) surface area of 285. 9 m^2^ g^−1^. Moreover, a sharp N_2_ uptake and an open hysteresis loop are observed at low relative pressures (P/P_0_ < 0.1), implying a disordered microporous structure derived from FA pyrolysis [48]. The corresponding pore size distributions are calculated using Barrett-Joyner-Halenda (BJH) method and the results in Fig. S6b show a hierarchical porous structure (dominantly micropores and mesopores) for Mn–N–C SAC. In the absence of Mn, N-doped carbon sheets (denoted as NC NS) are prepared (Fig. S7).

**Fig. 2:**
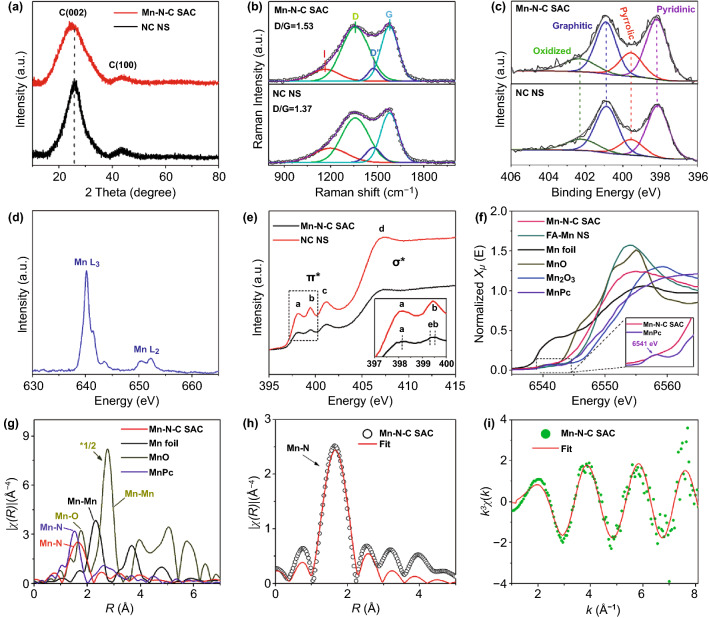
**a** PXRD patterns, **b** Raman spectra, **c** N 1s XPS spectra of Mn–N–C SAC and NC NS. **d** Mn L-edge XAS spectrum of Mn–N–C SAC. **e** N K-edge XAS spectra of Mn–N–C SAC and NC NS. **f** XANES of Mn–N–C SAC, FA-Mn NS and the standard references. Inset is the enlarged XANES spectra of Mn–N–C SAC and MnPc from the dashed rectangle area. **g** FT-EXAFS spectra of Mn–N–C SAC and the standard references. **h, i** FT-EXAFS curve fitting of Mn–N–C SAC in R and k space

Figure 2a shows the powder X-ray diffraction (PXRD) patterns of Mn–N–C SAC and NC NS, in which only C (002) and less prominent C (101) diffraction peaks are detected. The absence of diffraction peaks corresponding to Mn aggregates confirms a high dispersion of Mn atoms. Compared with NC NS, Mn–N–C SAC displays a broader and negatively shifted C (002) peak, implying that Mn doping makes the catalyst more defective and less graphitized. Raman spectra in Fig. 2b show an increased area ratio of peak D and peak G (*i.e.*, D/G) from 1.37 to 1.53 after Mn doping, indicating additional structural defects and a reduced extent of graphitization. Such structural change can partially disturb the electron transfer (*i.e.*, conductivity) over the catalysts, as being confirmed by a slight increase in series resistance in the electrochemical impedance spectra (Fig. S8). XPS is performed to further investigate the chemical and electronic states of Mn–N–C SAC. As expected, C, N, Mn, and O signals are detected and quantitively summarized in Fig. S9a. The high-resolution Mn 2p XPS spectrum in Fig. S9b shows Mn 2p_3/2_ and Mn 2p_1/2_ peaks at 641.40 and 653.28 eV, respectively, which are close to those of Mn^2+^ and thus suggest a valence state of + 2 for Mn atoms [49]. In Fig. 2c, both the N 1s spectra of Mn–N–C SAC and NC NS can be fitted into pyridinic N, pyrrolic N, quaternary N, and oxidized N with minute changes in their corresponding binding energy [27, 28, 50]. The detail fitting results can be found in Table S1. Compared with NC NS, the pyrrolic N of Mn–N–C SAC is increased by 4.0% and is likely contributed by the formed Mn–N_x_ moieties. Mn–N_x_ component is not fitted in detail here due to its near binding energy to that of pyrrolic N and pyridinic N [28, 29, 31, 51], as being further confirmed by the following N K-edge X-ray absorption spectroscopy (XAS) analysis. In addition, the percentage of quaternary N decreases by 4.2%, suggesting that the Mn–N coordination occupies the N sites for the formation of quaternary N.

More insightful atomic and electronic structures of the samples are analyzed by synchrotron-radiation-based XAS. Soft X-ray absorption near edge structure (XANES) analysis is firstly undertaken in a total electron yield (TEY) mode. In the Mn L_2,3_-edge spectrum (Fig. 2d), the L_3_ edge gives a dominant peak at 640.1 eV neighboured by two minor peaks at 641.3 and 643.5 eV, and the L_2_ edge presents two comparable peaks at 650.5 and 652.2 eV. These peaks are assigned to Mn 2p → Mn 3d transitions and are characteristics of ionic Mn^II^ systems [52]. The valence bonds of the catalyst are further examined by N K-edge spectra analysis (Fig. 2e). The spectrum of NC NS presents four nitrogen features: pyridinic (peak a, 398.2 eV), pyrrolic (399.4 eV), graphitic (peak c, 401.1 eV) and C–N σ^*^ bond (peak d, 407.3 eV), consisting with XPS results. The N K-edge of Mn–N–C SAC largely maintains the features of NC NS; however, a new peak e near to peak b is formed and assigned to Mn–N bond interaction, in accordance with previous studies [53, 54]. Nevertheless, the contributions of N species to Mn–N bonds are not resolvable in the experimental spectrum.

Mn K-edge XANES and phase-uncorrected Fourier transformed (FT) extended X-ray absorption fine structure (EXAFS) spectroscopy are further performed to analyze the chemical and coordination structures of Mn–N–C SAC. The reference standards including Mn foil, MnO, Mn_2_O_3_ and Mn phthalocyanine (MnPc) together with the precursor FA-Mn NS are also characterized for comparison. For the Mn K-edge XANES curves in Fig. 2f, both the absorption edge and white line peak of Mn–N–C SAC is located close to those of Mn (II) in FA-Mn NS and Mn (II) in MnO, but are far from those of Mn (III) Mn_2_O_3_, indicating that the valence of Mn species in Mn–N–C SAC is close to + 2. It is consistent with Mn L-edge results. MnPc has a perfect Mn–N_4_ square planar symmetry and such D4h centrosymmetric coordination gives a pre-edge feature at 6541 eV in the plotted XANES spectra (Fig. 2f). The pre-edge peak is generally assigned to the dipole-forbidden 1 s → 3d transition for transition metals [46]. Compared with MnPc, Mn–N–C SAC exhibits a more evenly increased signal intensity in the pre-edge region, implying a different local coordination structure of the atomic Mn sites. From the EXAFS curves in Fig. 2g, it can be observed that Mn–N–C SAC gives a nearly symmetric dominant peak at 1.66 Å. Considering the formed Mn–N structures in the N K-edge XANES spectrum, the peak is assigned to Mn–N scattering path. Unlike Mn foil and MnO, no obvious peaks for Mn–Mn scattering path at 2.32 and 2.76 Å are observed, corroborating the homogeneous dispersion of Mn atoms over the ultrathin carbon sheets [29, 31]. The EXAFS spectrum of MnPc presents a main peak at 1.52 Å, which is assigned to the scattering of Mn–N (Fig. 2g). Compared to MnPc, the larger Mn–N distance for Mn–N–C SAC implies a different Mn–N coordination structure from the symmetrical Mn–N_4_. The structural parameters are obtained from the quantitative least-square EXAFS curve fitting in R and k spaces (Fig. 2h, i and Table S2). The results show that the fitting curves match satisfactorily with the experimental data. The EXAFS fitting analysis for standard MnO is also given in Fig. S10. The Mn–N scattering path in Mn–N–C SAC displays an average coordination number of 2.7 ± 0.2, indicating that the isolated Mn atom likely gives a three-fold Mn–N_3_ coordination structure. The Mn–N interaction gives a mean bond length of 2.19 Å, longer than those of MnPc (1.94 Å) with square planar Mn–N_4_ structure, Mn SAC with Mn–N_4_ structure (1.95 Å) [31] and Mn SAC with Cl-Mn–N_4_ structure (2.08 Å) [27]. These structural features evidence a different Mn–N coordination environment from the typical Mn–N_4_ coordination. The results verify the formation of Mn SACs with massive atomic Mn sites being homogeneously distributed on ultrathin carbon sheets.

### Electrochemical Performance of Mn–N–C SAC for NRR

**Fig. 3:**
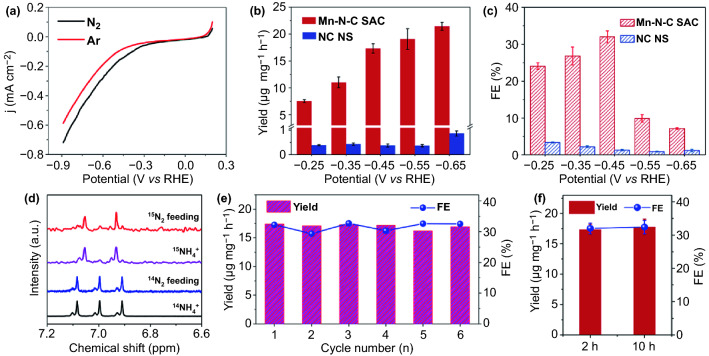
**a** LSV curves of Mn–N–C SAC obtained at a scan rate of 10 mV s^−1^ under N_2_ and Ar environment. **b** NH_3_ yields and **c** FEs of Mn–N–C SAC and NC NS at various potentials. **d** ^1^H-NMR spectra of the electrolytes using ^15/14^N_2_ as feedstock gases. **e** NH_3_ yields and FEs of Mn–N–C SAC at  − 0.45 V after independently repetitive electrocatalysis experiments. **f** FEs and yields for the 10-h durability test at -0.45 V

The NRR activities of the catalysts are evaluated using a carbon fiber paper electrode in 0.1 M NaOH. All potentials are referred to reversible hydrogen electrode (RHE). Figure 3a shows the linear sweep voltammetry (LSV) curves of Mn–N–C SAC. Evidently, the current density in N_2_-saturated electrolyte is larger than that in Ar-saturated electrolyte over a wide potential range from -0.2 to -0.9 V, implying that Mn–N–C SAC is active for NRR. To quantify the NRR activity, chronoamperometry electrocatalyses are conducted. NC NS is also measured for comparison. The produced ammonia is detected by indophenol blue method with the calibration curve being provided in Fig. S11. The average NH_3_ yield rates and Faradaic efficiencies (FEs) of Mn–N–C SAC and NC NS at different potentials are calculated from the obtained chronoamperometric curves and the corresponding UV–vis spectra in Figs. S12 and S13. As shown in Fig. 3b, the NH_3_ yield rate of Mn–N–C SAC increases with the cathodic potential becoming more negative and reaches up to 21.43 μg h^−1^ mg^−1^_cat._ at -0.65 V. By contrast, the NC NS counterpart exhibits a negligible NH_3_ yield rate at all applied potentials (only 0.87 μg h^−1^ mg^−1^_cat._ -0.65 V). This corroborates the critical role of Mn–N_3_ moieties for NRR. In addition, Mn–N–C SAC delivers a substantial higher FEs for NH_3_ production than NC NS at all potentials (Fig. 3c). Remarkably, the NH_3_ FE of Mn–N–C SAC reaches up to 32.02% at -0.45 V, which is *ca.* 25-fold higher than that of NC NS. It is observed that NC NS gives a higher current density than Mn–N–C SAC (Figs. S12 and S13), which is likely contributed by the remarkable HER activity and increased graphitization degree (as evidenced by XRD and Raman). Beyond -0.45 V, the NH_3_ FE of Mn–N–C SAC experiences a significant decrease as a result of the accelerated HER. The NH_3_ yield rate and FE values make Mn–N–C SAC among the best NRR electrocatalysts to date (Table S3).

Hydrazine is often a competitive product of NRR. In this work, no hydrazine is detected by Watt-Chrisp method (Fig. S14), verifying the excellent selectivity of Mn–N–C SAC toward NH_3_. To exclude the possible NH_3_ interferences, a set of control experiments are further performed. As evidenced by the corresponding UV–vis spectra (Fig. S15), no NH_3_ is detected from the electrolytes of Mn–N–C SAC-based electrolysis system while using Ar as feeding gas or operating at the open-circuit condition. Moreover, bare carbon fiber paper is measured to be inert for NRR (Fig. S15). The results reflect a reliable NH_3_ production data deriving from NRR. The origination of NH_3_ is further verified by isotope-labeled experiment, in which the ammonia-containing electrolyte is monitored by ^1^H NMR (Fig. 3d). Evidently, a double of notable peaks with a coupling constant of 72 Hz is detected using ^15^N_2_ (^15^ N≡^15^ N, 99%) as the N source, which match well with those of ^15^(NH_4_)_2_SO_4_ standard reference. While ^14^N_2_ feeding gas being used, the triple signal with a coupling constant of 52 Hz is measured, which is identical to that of ^14^(NH_4_)_2_SO_4_ standard sample. The results confirm the generation of NH_3_ from NRR. Stability is another important criterion for NRR electrocatalysts. As depicted in Figs. 3e and S16, six successive recycling tests at -0.45 V lead to no obvious fluctuation of NH_3_ yield rate and FE for Mn–N–C SAC. Further, the catalyst exhibits no decrease in these values even after being subjected to a 10 h testing period (Figs. 3f and S17). The results suggest the strong stability of Mn–N–C SAC for NRR.

### NRR Mechanism Analysis

**Fig. 4:**
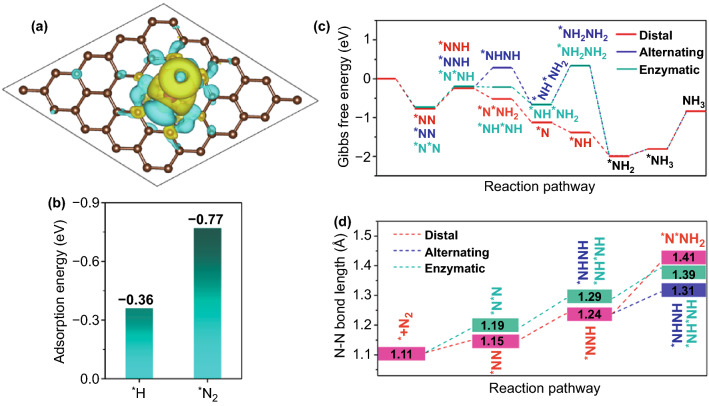
**a** Charge differential distribution of Mn–N–C SAC after the adsorption of N_2_ via end-on mode. **b** Adsorption energy of *N_2_ and *H on Mn site. **c** Gibbs free energy diagrams of distal, alternating and enzymatic pathways for N_2_ reduction, and **d** the corresponding N–N bond length of the intermediates

DFT studies are performed to fundamentally understand the NRR process on Mn–N–C SAC. According to the calculation results, Mn–N_3_ site can effectively adsorb N_2_ via both end-on and side-on patterns with a similar adsorption energy of -0.77 and -0.74 eV, respectively. Bader charge analysis on a end-on adsorption mode shows the electron back-donation (0.48 e^−^) from Mn atom to the adsorbed N_2_ (Fig. 4a), which indicates the favored activation of N_2_ for subsequent hydrogenation [55]. The selectivity toward N_2_ and H adsorption is a key metric for NRR catalysts. As shown in Fig. 4b, Mn–N_3_ site presents a more negative adsorption energy for N_2_ than that for H, indicating sufficient N_2_ binding at the potential of 0 V versus normal hydrogen electrode (NHE). This is expected to favor a less hindered N_2_ adsorption by H adsorption at low overpotential [10, 16].

To determine the reaction pathway of NRR on Mn–N–C SAC, all the possible intermediates with both end-on and side-on adsorbed configurations are considered (Fig. S18 and Tables S4 and S5). Figure 4c depicts the corresponding energy profiles. Different from the situation of other electrocatalyst, Mn–N–C SAC displays an optimal side-on adsorption configuration toward N-NH_2_ in the distal pathway, *i.e.,* *N*NH_2_. Such bonding configuration, compared to *NHNH in the alternating pathway and *NH*NH in the enzymatic pathway, exhibits a more negative free-energy variation (ΔG) while evolving from NNH. As such, NH_3_ synthesis on Mn–N–C SAC prefers to proceed by the distal mechanism. The first protonation step of N_2_ (*i.e.*, *N_2_ → *NNH) exhibits the highest energy barrier in this reaction process, thus occurring as the potential determining step (PDS) with ΔG_PDS_ of 0.53 eV. Despite being a thermodynamically unfavorable step, the ΔG_PDS_ is much smaller than those of N–N bond cleavage energy without the catalyst (4.26 eV) [56] or on the benchmark Ru (0001) stepped surface (0.98 eV) [18], suggesting the superior catalytic activity of Mn–N–C SAC. The desorption of *NH_3_ in the last step is not considered here due to the significantly accumulated free energy (-1.81 eV) in previous reaction processes [57]. The ΔG for hydrazine formation on Mn–N–C SAC reaches a prohibitively high value of 1.00 eV. This agrees well with the absence of hydrazine in our experimental measurements. Lastly, the N–N bond length analysis is performed to further understand the catalytic effect of Mn–N–C SAC (Fig. 4d). In the distal pathway, the Mn active site can properly activate the adsorbed N_2_ by stretching the N–N bond length from the initial 1.10 to 1.19 Å. The N–N bond length experiences a nearly linear elongation and reaches to 1.41 Å for *N*NH_2_, which is larger than that of *NHNH (1.39 Å) and *NH*NH (1.31 Å). The increased bond length favors the easy cleavage of the N–N bond in a distal mechanism. Therefore, we conclude that Mn–N–C SAC with Mn–N_3_ active sites is satisfied for NRR in both selectivity and catalytic activity.

## Conclusions

In summary, we demonstrate a facile folic acid self-assembly strategy for the fabrication of Mn–N–C SAC, which has a high Mn loading content of 2.82 wt% and a homogeneous atomistic metal dispersion on well-exposed carbon sheets. The Mn–N–C SAC achieves a high NH_3_ FE of 32.02% and a desirable yield rate of 21.43 μg h^−1^ mg^−1^_cat._ in 0.1 M NaOH. DFT calculations unveil the critical role and catalytic mechanism of atomic Mn sites toward NRR. Remarkably, the atomic Mn sites can significantly promote the adsorption of reacting intermediates and reduce the energy barrier of the first hydrogenation step for efficient NRR. Our work provides a new strategy for the rational fabrication of Mn SAC and expands the catalyst to effective NRR.

## Supplementary Information

Below is the link to the electronic supplementary material.

Supplementary file1 (PDF 1672 kb)
