# Supplementary material for: Folic Acid Self-Assembly Enabling Manganese Single-Atom Electrocatalyst for Selective Nitrogen Reduction to Ammonia
Source: Nanomicro Lett. 2021 May 12;13:125. doi: 10.1007/s40820-021-00651-1 (PMC8113419; doi:10.1007/s40820-021-00651-1)
Supplement: Supplementary file 1 — Supplementary file1 (PDF 1672 kb) [file 40820_2021_651_MOESM1_ESM.pdf]

Nano-Micro Letters

Supporting Information for

Folic Acid Self-Assembly Enabling Manganese Single-Atom Electrocatalyst for Selective Nitrogen Reduction to Ammonia

Xuewan Wang[1], Dan Wu[1], Suyun Liu[1], Jiujun Zhang[2], Xian-Zhu Fu[1, *], Jing-Li Luo[1, *]

[1]Shenzhen Key Laboratory of Polymer Science and Technology, Guangdong Research Center for Interfacial Engineering of Functional Materials, College of Materials Science and Engineering, Shenzhen University, Shenzhen 518060, P. R. China

[2]Institute for Sustainable Energy, College of Sciences, Shanghai University, Shanghai, 200444, P. R. China

*Corresponding authors. E-mail: xz.fu@szu.edu.cn (Xian-Zhu Fu); jingli.luo@ualberta.ca (Jing-Li Luo)

Supplementary Figures and Tables

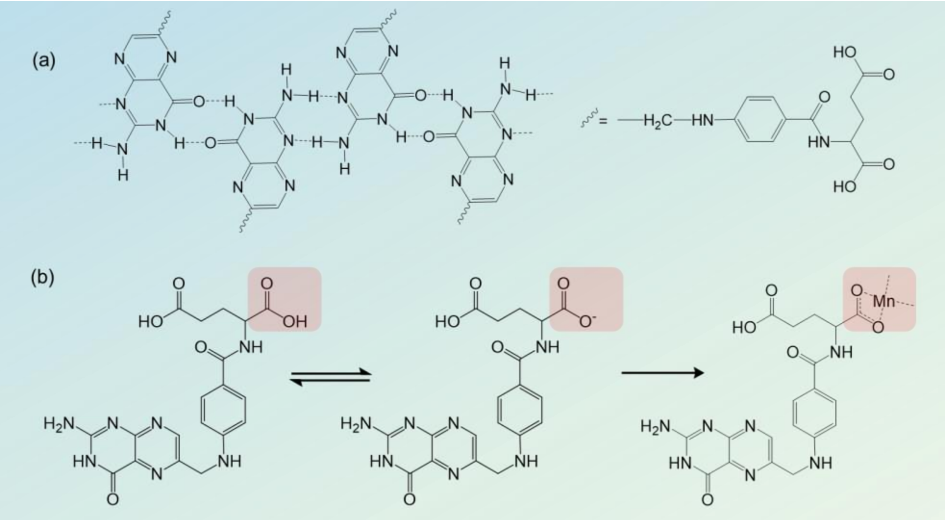

Fig. S1 a Schematic of the FA self-assembly *via* complementary hydrogen bonding at pteridine group. **b** The partial dissociation of FA molecule at α-carboxyl group and its chelating property towards Mn^{2+}

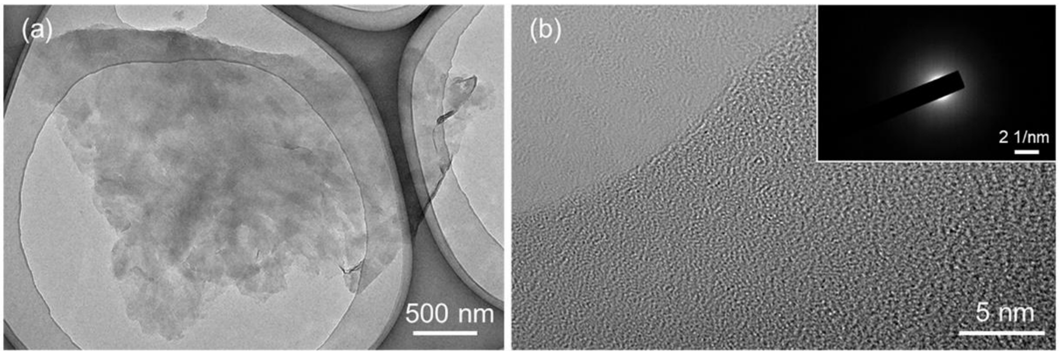

Fig. S2 a, b HR-TEM images of FA-Mn NS. Inset in **b** is the selected area diffraction pattern of FA-Mn NS

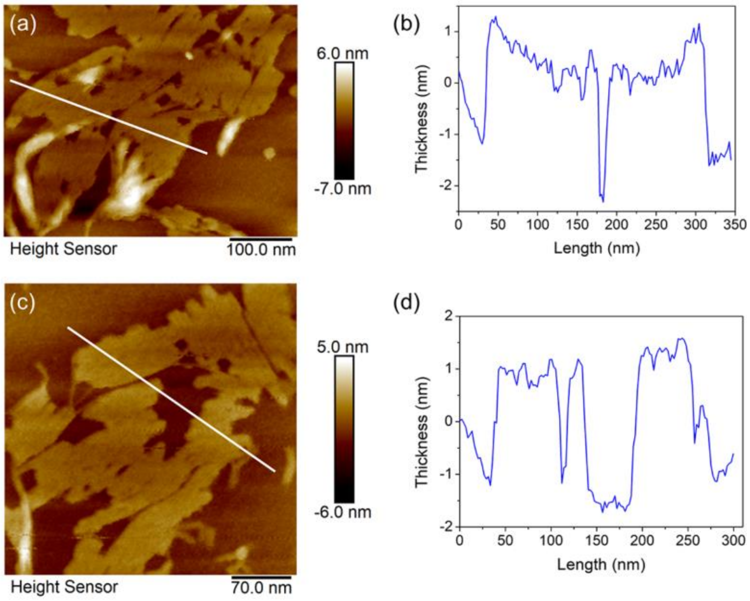

Fig. S3 a, c AFM images of FA-Mn NS, and **b, d** the corresponding topography line profiles

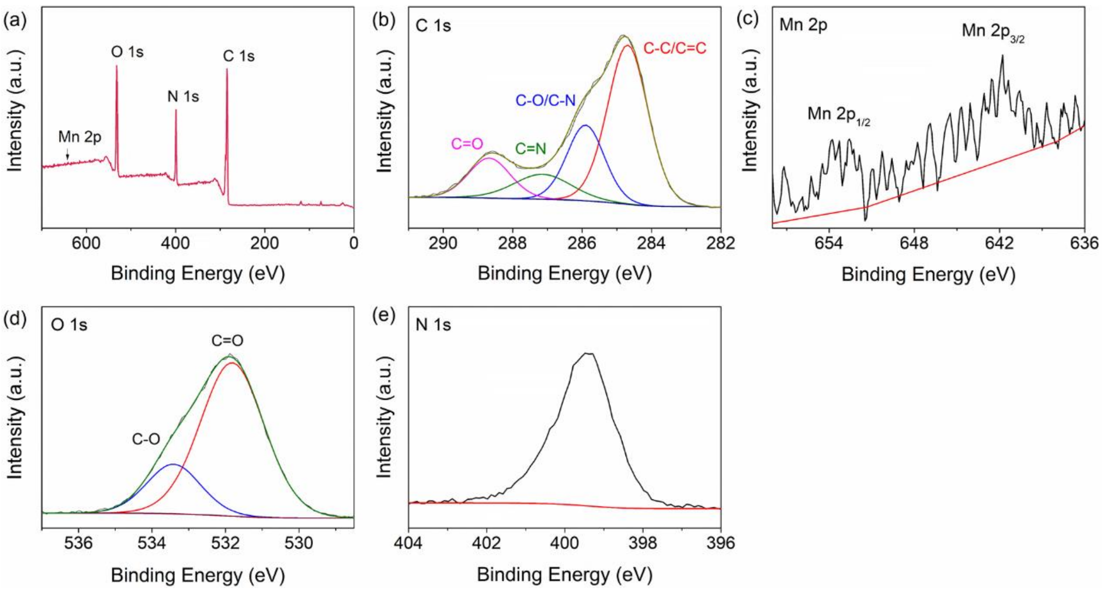

Fig. S4 a XPS survey, **b** C 1s b, **c** Mn 2p, **d** O 1s and **e** N 1s for FA-Mn NS. The C 1s spectrum for Mn-FA NS consist of four components corresponding to C-C/C=C (284.7 eV), C-O/C-N (285.9 eV), C=N (287.1 eV) and C=O (288.7 eV) species. The O 1s spectrum is fitted into two configurations, including C=O (531.8 eV) and C-O (533.4 eV). The N 1s spectrum was not fitted in detail here. Minor Mn 2p signal was measured due to a low content of Mn^{2+}. The data was analyzed by referring to the data from https://xpssimplified.com/elements/carbon.php. All the components are consistent with the functional groups of FA, suggesting the formation of FA-Mn NS.

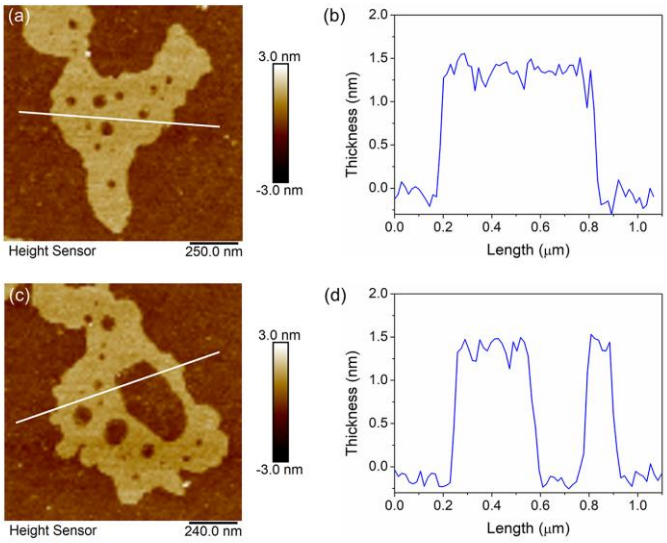

Fig. S5 a, c AFM images of Mn-N-C SAC nanosheets, and **b, d** the corresponding topography line profiles

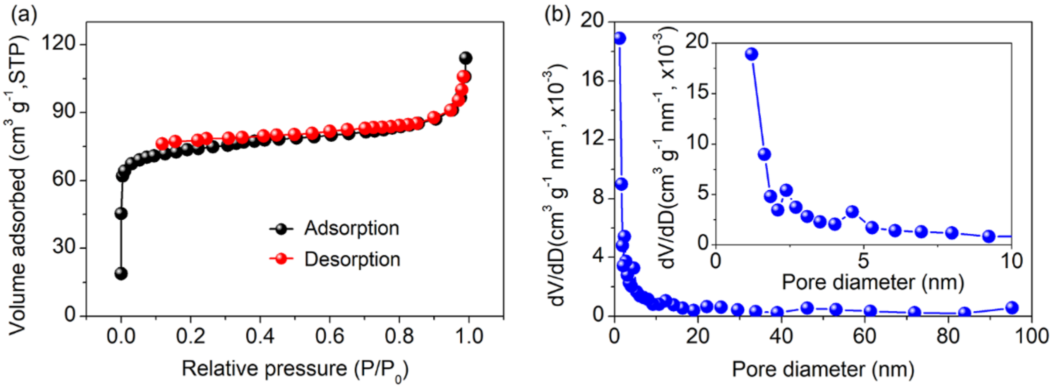

Fig. S6 a N_2 adsorption-desorption isotherms of Co-N-C SAC and **b** the corresponding pore size distribution curve. Inset in **b** is the magnified pore size distribution in a pore diameter range of 0-10 nm.

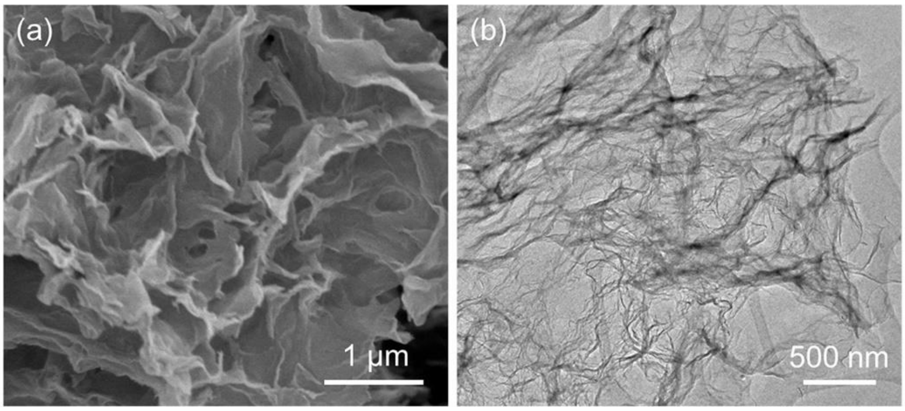

Fig. S7 a SEM and **b** TEM images of NC NS

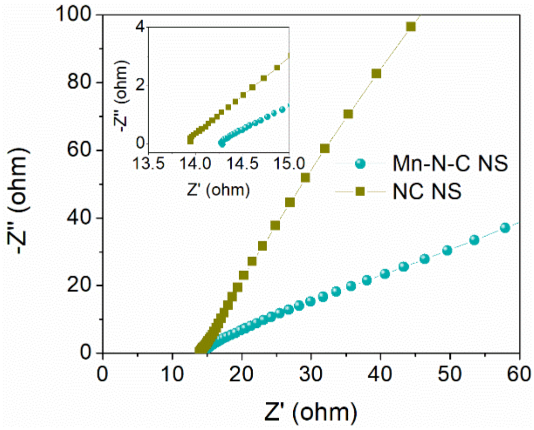

Fig. S8 Nyquist plots of Mn-N-C SAC and NC NS, which are measured on carbon fiber electrodes in H-type cell and at a potential of 0 V versus RHE

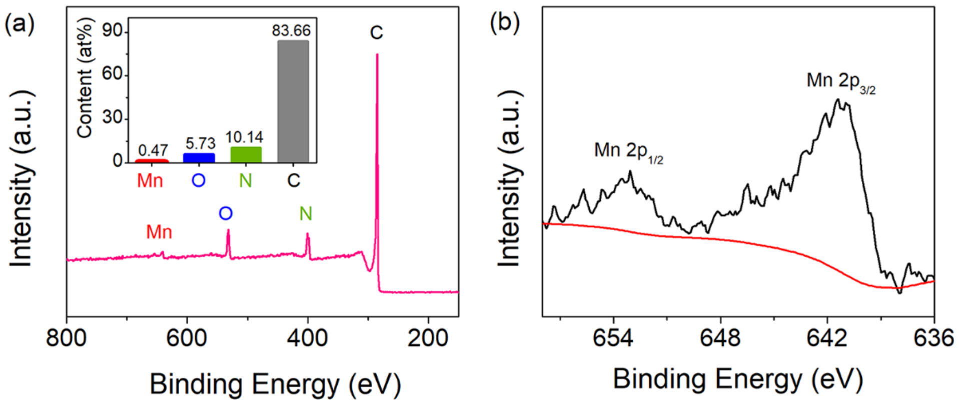

Fig. S9 a XPS survey spectrum of Mn-N-C SAC. Inset is the content of the measured components. **b** High resolution Mn 2p XPS spectra.

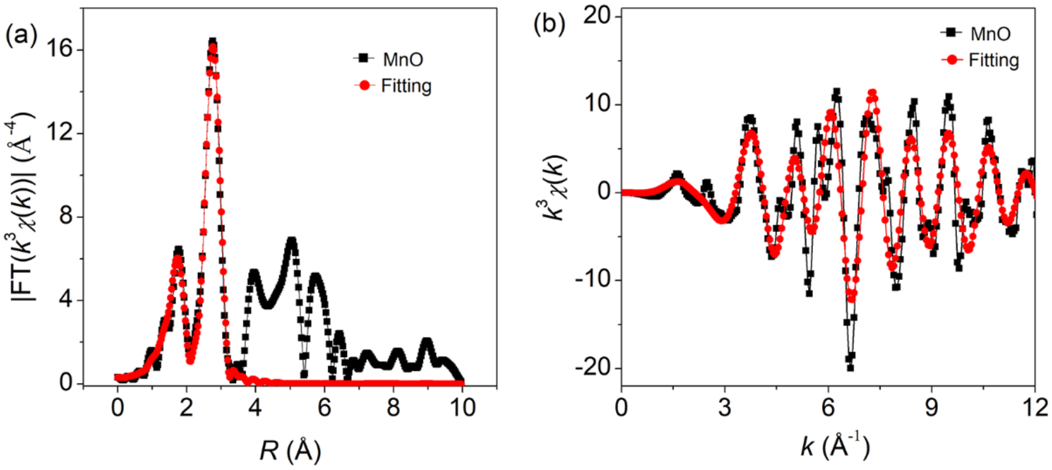

Fig. S10 FT-EXAFS curve fitting of MnO in **a** R space and **b** k space

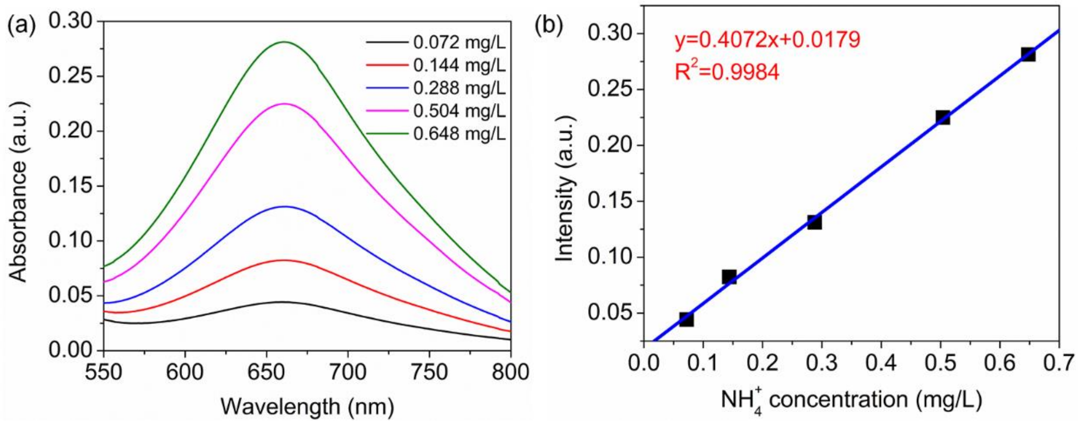

Fig. S11 a UV-vis spectra for standard NH_4^+ solution in 0.1 M NaOH with different concentrations and **b** the corresponding calibration curve

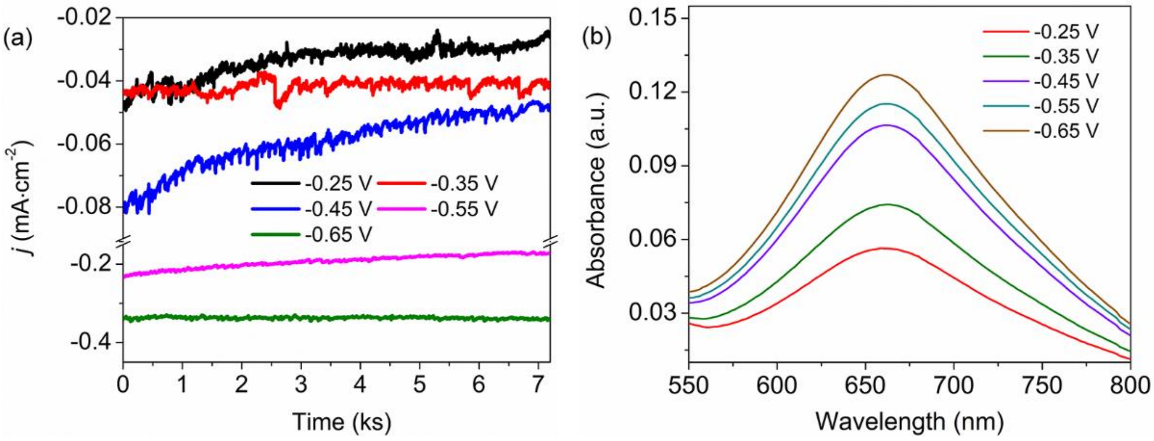

Fig. S12 a Chronoamperometry curves of Mn-N-C SAC at different potentials and **b** the corresponding UV-Vis absorption curve of the produced electrolyte measured by indophenol blue method

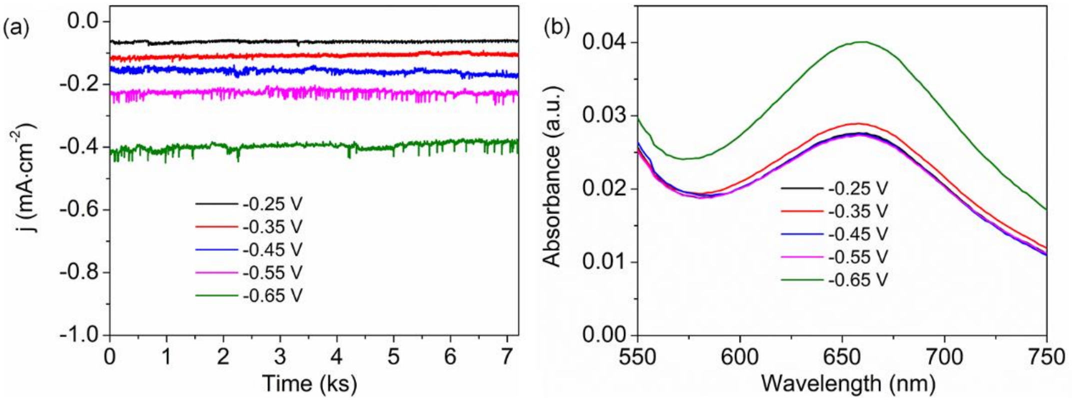

Fig. S13 a Chronoamperometry curves of NC NS at different potentials and **b** the corresponding UV-Vis absorption of the produced electrolyte measured by indophenol blue method

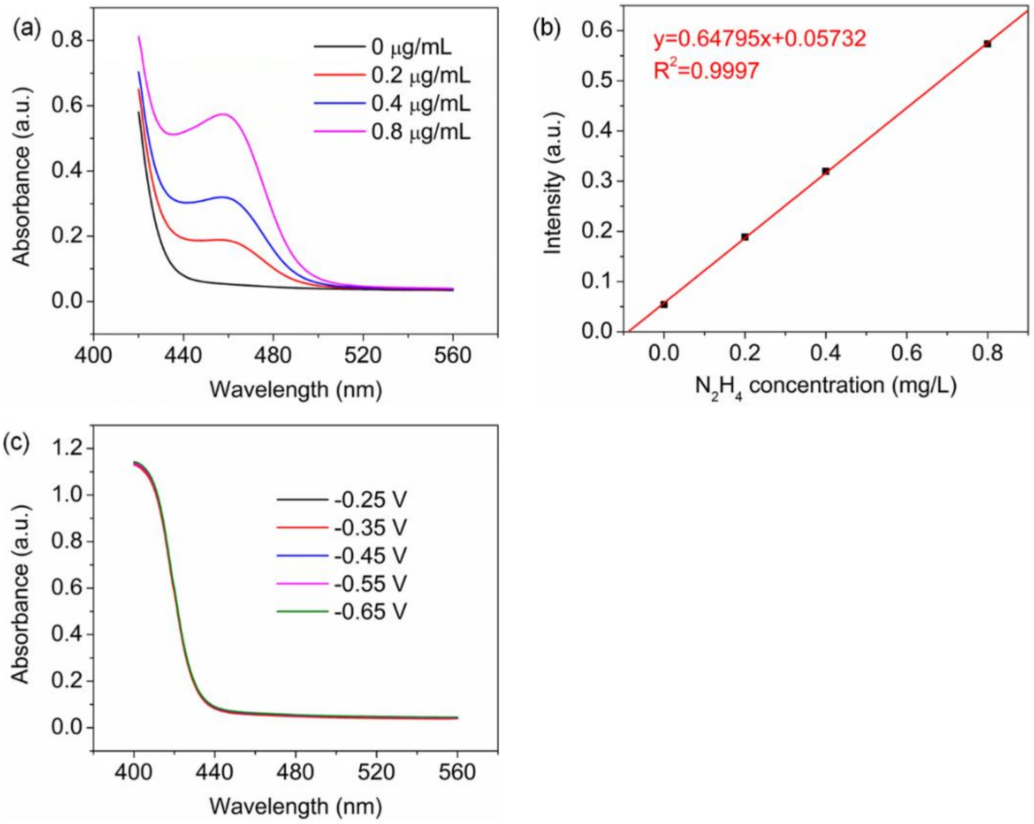

Fig. S14 a UV-vis spectra for the standard hydrazine hydrate in 0.1 M NaOH and **b** the corresponding calibration curve. **c** UV-vis spectra for the electrolyte obtained from Mn-N-C SAC after the electrolysis at different potentials. It is evident that no hydrazine is detected in this work.

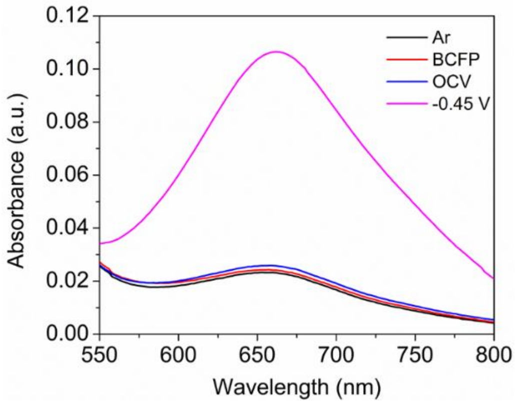

Fig. S15 UV-Vis spectra of the electrolyte obtained from the Mn-N-C SAC-based electrolysis system using Ar as the feeding gas (black line), from the Mn-N-C SAC-based electrolysis system measured at open-circuit potential (OCV, blue line), from the Mn-N-C SAC-based electrolysis system measured using N_2 as the feeding gas at -0.45 V (pink line) and from the bare carbon fiber paper (BCFP)-based electrolysis system using N_2 as the feeding gas (red line).

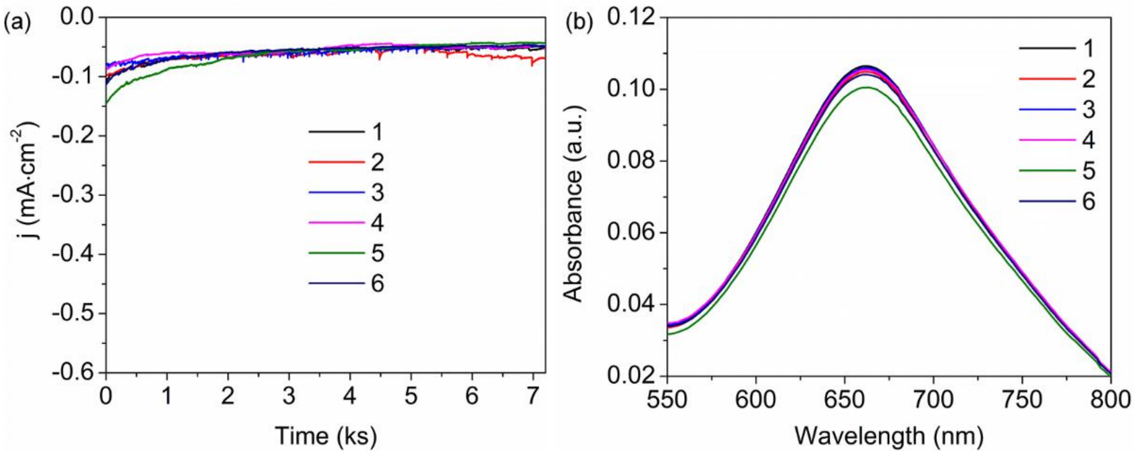

Fig. S16 a Chronoamperometry curves of Mn-N-C SAC for six repetitive electrocatalysis measurement and **b** the corresponding UV-Vis absorption of the produced electrolyte measured by indophenol blue method

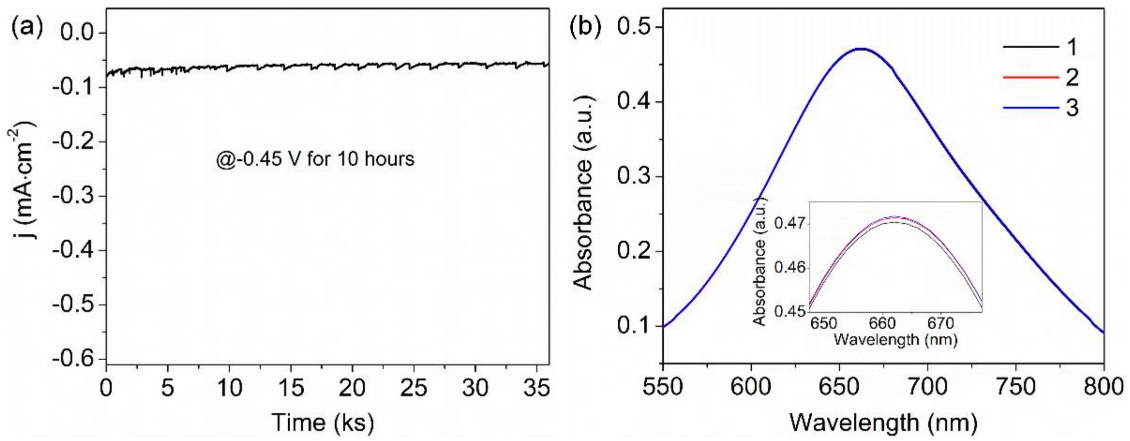

Fig. S17 a Chronoamperometry curves of Mn-N-C SAC for the 10-hour durability test at the optimal potential of -0.45 V and **b** the corresponding UV-Vis absorption of the produced electrolyte measured by indophenol blue method

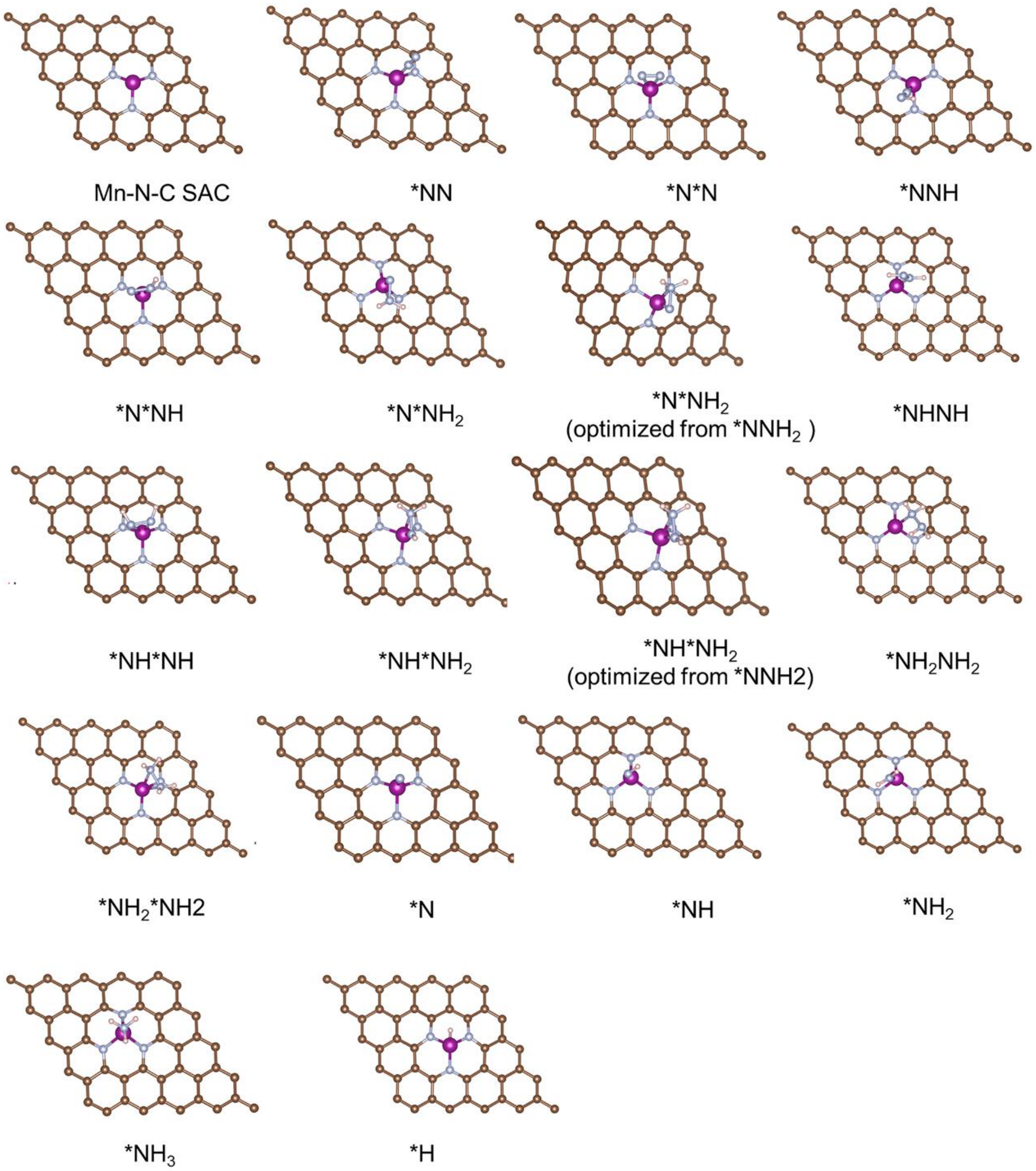

Fig. S18 Optimized bonding configurations of the various intermediates on Mn-N-C SAC

Table S1 The fitting results of N 1s XPS spectra for Mn-N-C SAC and NC NS (%)

	Pyridinic N	Pyrrolic N	Quaternary N	Oxidized N
Mn-N-C SAC	38.8	16.3	32.3	12.6
C NS	38.7	12.3	36.5	12.5

Table S2 EXAFS fitting parameters at the Mn K-edge for various samples (S_0^2=0.73)

Sample	shell	CN	R(Å)	σ^2	ΔE_0	R factor
MnO	Mn-O	6	2.19±0.01	0.0077	0.4±0.8	0.0071
	Mn-Mn	6	3.19±0.03	0.0048		
	Mn-Mn	6	3.08±0.02			
Mn-N-C SAC	Mn-N	2.7±0.2	2.19±0.02	0.0031	5.1±2.1	0.0118

Table Footnote: *CN*: coordination numbers; *R*: bond distance; σ^2: Debye-Waller factors; ΔE_0: the inner potential correction. *R* factor: goodness of fit. S_0^2 was set to 0.73, according to the experimental EXAFS fit of MnO reference by fixing CN as the known crystallographic value; δ: percentage.

Table S3 Performances of the recently reported state-of-the-art NRR SACs, representative other types of electrocatalysts and Mn-N-C SAC

**Catalyst**	**Electrolyte**	**NH_3 Yield Rate**	**Faradaic Efficiency**	**References**
Mn-N-C SAC	0.1 M KOH	21.43 μg h^{-1} mg^{-1}_{cat}	32.02%	**This work**
Ru SAC/g-C_3N_4	0.5 M NaOH	23.0 μg h^{-1} mg^{-1}_{cat}	8.3%	Adv. Funct. Mater. 2020, 30, 1905665.
Fe-(O-C_2)$_4$ SAC	0.1 M KOH	32.1 μg h^{-1} mg^{-1}_{cat}	29.3%	Angew. Chem. Int. Ed. 2020, 59, 13423
Mo SAC/NC	0.1 M KOH	34.0±3.6 μg h^{-1} mg^{-1}	14.6±1.6%	Angew. Chem. Int. Ed., 2019, 58, 2321
Fe_{SA}-N-C	0.1 M KOH	7.48 μg h^{-1} mg^{-1}	56.66%	Nat. Commun. 2019, 10, 341
Cu-N-C SAC	0.1 M KOH	53.3 μg h^{-1} mg^{-1}	13.8%	ACS Catal. 2019, 9, 10166
Cu NP/Polyimide	0.1 M KOH	2.48 μg h^{-1} mg^{-1}	6.56%	Nat. Commun., 2019, 10, 4380
MnO_3N_1/PC	0.1 M HCl	66.41 μg h^{-1} mg^{-1}	8.91%	ACS Catal., 2021, 11, 509
Ru SAC/NC	0.05 M H_2SO_4	120.9 μg h^{-1} mg^{-1}	29.6%	Adv. Mater., 2018, 30, 1803498
Co-N-C SAC	0.005 M H_2SO_4	16.9μg h^{-1} mg^{-1}_{cat}	18.9%	ACS Appl. Energy Mater. 2020, 3, 6079
Ru SAC/Mo_2CT_x MXene	0.5 M K_2SO_4	40.57 μg h^{-1} mg^{-1}_{cat}	25.77%	Adv. Energy Mater., 2020, 10, 2001364
Ru@ZrO_2/NC	0.1 M HCl	3.7 μg h^{-1} mg^{-1}	21.0%	Chem-Us., 2019, 5, 204
Mo SAC-Mo_2C/NCNT	0.005 M H_2SO_4 + 0.1 M K_2SO_4	16.1 μg h^{-1} mg^{-1}_{cat}	7.1%	Adv. Mater., 2020, 32, 2002177
B-doped graphene	0.05 M H_2SO_4	9.8 μg h^{-1} cm^{-2}	10.8%	Joule, 2018, 2, 1610

Carbon nitride	0.1 M HCl	2.9 μg h^{-1} mg^{-1}	16.8%	Nano Lett., 2020, 20, 2879-2885
Au_4Pt_2/graphene	0.1 M HCl	7.9 μg h^{-1} mg^{-1}	9.7%	Nat. Commun. 2020, 11, 4389
Ru NP/rGO	0.05 M H_2SO_4	50 μg h^{-1} mg^{-1}	11%	Angew. Chem. Int. Ed., 2020, 132, 21465
MXene/$TiFeO_x$	0.05 M H_2SO_4	21.90 μg h^{-1} mg^{-1}	25.44%	Acs Nano, 2020, 14, 9089
CoS_x/NS-G	0.05 M H_2SO_4	25.0 μg h^{-1} mg^{-1}	25.9%	Proc. Natl. Acad. Sci., 2019, 116, 6635
MoS_2	0.1 M Na_2SO_4	4.93 μg h^{-1} cm^{-2}	1.17%	Adv. Mater. 2018, 30, 1800191
Fe-doped $W_{18}O_{49}$	0.25 M $LiClO_4$	24.7μg h^{-1} mg^{-1}	20%	Angew. Chem. Int. Ed., 2020, 59, 7356
CNT@g-C_3N_4-FeCu NC	- $LiClO_4$	9.86 μg h^{-1} mg^{-1}	34%	Adv. Mater., 2020, 32, 2004382

Table S4 The obtained energy values of the intermediates from DFT calculations

adsorbate	E_{tot} (eV)	G_{corr} (eV)	G (eV)
*	-455.871	0.000	-455.871
*NN	-473.703	0.111	-473.592
*N*N	-473.646	0.079	-473.567
*NNH	-476.867	0.400	-476.467
*N*NH	-476.823	0.393	-476.430
NNH_2	-480.940	0.785	-480.155
*NHNH	-480.035	0.693	-479.342
*NH*NH	-480.531	0.686	-479.845
NHNH_2,	-484.750	1.055	-483.695
*NH_2NH_2	-487.462	1.366	-486.096
*NH_2*NH_2,	-487.480	1.384	-486.096
*N	-465.107	0.051	-465.056
*NH	-469.006	0.293	-468.713
*NH_2	-473.332	0.613	-472.719
*NH_3	-476.856	0.910	-475.946
*H	-459.792	0.164	-459.628

Table S5 The obtained energy values of the corresponding molecules from DFT calculations

molecule	E_{tot} (eV)	G_{corr} (eV)	G (eV)
N_2	-16.604	-0.352	-16.956
H_2	-6.758	-0.046	-6.804
NH_3	-19.518	0.417	-19.101
